# Autism Spectrum Traits in Children with Anxiety Disorders

**DOI:** 10.1007/s10803-012-1575-z

**Published:** 2012-06-26

**Authors:** Francisca J. A. van Steensel, Susan M. Bögels, Jeffrey J. Wood

**Affiliations:** 1 Research Institute of Child Development and Education, University of Amsterdam, Nieuwe Prinsengracht 130, 1018 VZ Amsterdam, The Netherlands; 2 Departments of Education and Psychiatry and Biobehavioral Sciences, University of California, Los Angeles, CA 90095 USA

**Keywords:** Anxiety, ASD, ADI-R, Children, Risk factor

## Abstract

The aim of this study was to examine ASD traits in children with clinical anxiety in early development, as well as current manifestations. Parents of 42 children with an anxiety disorder (but no known diagnosis of ASD) and 42 typically developing children were interviewed using the Autism Diagnostic Interview (ADI-R). They also completed questionnaires that assessed child anxiety (SCARED-71) and children’s ASD symptoms. Results revealed that children with anxiety disorders had higher scores than typically developing children, for both ASD traits in early development as well as current ASD symptoms. A specific association was found between symptoms of Social Anxiety Disorder and ASD traits early in life. Findings are considered in terms of clinical implications, and limitations are discussed.

## Introduction

To respond with anxiety to a threatening situation or stimuli is normal and healthy; however, when such a reaction becomes excessive and impairs daily functioning, one is classified as having an anxiety disorder (APA 2000). Anxiety disorders are among the most common disorders in childhood, with prevalence rates between 8.3 and 20.9 % (Costello et al. 2005). In addition, comorbidity among anxiety disorders as well as with other DSM-IV disorders is substantial (e.g., Kendall et al. 2010). Symptoms of anxiety disorders may include preoccupations and repetitive behaviors (e.g., obsessions and compulsions), avoidance of (social) situations, and speech problems (e.g., dysfluency), which are also commonly seen in children with autism spectrum disorders (ASD) (Hartley and Sikora 2009; Wood and Gadow 2010). ASD is characterized by impairments in three domains; (a) the social domain (e.g., impairments in the use of nonverbal behaviors, lack of sharing, lack of social or emotional reciprocity), (b) the communication domain (e.g., impairments in the ability to initiate or sustain a conversation, stereotyped and repetitive language), and (c) the domain of repetitive, stereotyped interests and behaviors (e.g., preoccupations, nonfunctional routines or rituals) (APA 2000). In addition, prevalence rates of anxiety disorders in youth with ASD are much higher (nearly 40 % estimated by a recent meta-analysis; van Steensel, Bögels and Perrin 2011) compared to those found in typically developing children, and the differentiation between ASD and anxiety disorder symptoms, particular in the case of obsessive compulsive disorder and social anxiety disorder, can be difficult.

A study of Hartley and Sikora (2009) explored which criteria effectively discriminate ASD from anxiety. They administered a semi-structured interview of DSM-IV-TR criteria for autistic disorder to the parents of children with ASD, children with anxiety disorders, and children with ADHD. In this study it was found that the domain of restricted/repetitive/stereotyped patterns could not discriminate between children with ASD and children with anxiety disorders, while the best differentiation between children with anxiety and children with ASD could be made through the communication domain. Within the social relatedness domain it was found that the children with ASD had higher endorsements of impaired non-verbal behavior and lack of seeking to share compared to the children with anxiety disorders, however, social/emotional reciprocity was a poor indicator for ASD (Hartley and Sikora 2009). In addition, recent studies have found elevated scores of ASD in children with anxiety and/or mood disorders (Towbin et al. 2005; Pine et al. 2008). The study of Towbin et al. (2005) examined ASD traits in a sample of children with mood and anxiety disorders with three ASD measures, namely the Social Communication Questionnaire (SCQ; Berument et al. 1999), the Social Responsiveness Scale (SRS; Constantino et al. 2000), and the Children’s Communication Checklist (CCC-2; Bishop 1998). In this study, 48.0 % of the sample scored in the ASD range on at least one measure. A subsequent study of Pine et al. (2008) examined ASD scores in youths with anxiety and/or mood disorders. The same three instruments were used to assess ASD; however, in this study a comparison group of healthy participants was added. Results revealed that 4.0–24.6 % of the clinically anxious children scored above ASD thresholds depending on which measurement was used (note that of the children with mood disorders these percentages ranged between 7.4 and 75.0 %). In addition, it was found that children with anxiety and/or mood disorders had significantly higher ASD scores compared to the children in the healthy sample (Pine et al. 2008).

In sum, research so far has found (a) high prevalence rates of anxiety disorders in children with ASD (e.g., van Steensel et al. 2011), (b) several similarities (and differences) between children with ASD and children with anxiety disorders with respect to the DSM-IV-TR-criteria of autistic disorder (Hartley and Sikora 2009), and (c) elevated scores on ASD measures in children with anxiety and/or mood disorders (Towbin et al. 2005; Pine et al. 2008). What is not yet known is whether these ASD-like behaviors in children with anxiety disorders (and mood disorders) are precursors of the anxiety disorders or not. Do current anxiety disorders manifest, in part, as ASD-like behavior (but possibly representing methodological artifact or phenocopy rather than true ASD), or, alternatively (or additionally) do ASD symptoms in some children with anxiety disorders begin early in life, consistent with the clinical course of true ASD, and perhaps cause anxiety and mood disorders later in childhood via stress generation (see Wood and Gadow 2010). Of note, the study of Hallett et al. (2010) examined the relationship between autistic-like and internalizing traits in children from the normal population using a longitudinal design. The authors found evidence for an asymmetric bi-directional relation between the two; autistic-like traits measured at age 7 contributed to internalizing traits measured at age 12 and vice versa (although the latter relation—early internalizing traits contributing to latter autistic-traits—was found to be somewhat smaller compared to the first; Hallett et al. 2010).

The aim of this study was to compare children with anxiety disorders to typically developing children with respect to current ASD-like behaviors as well as ASD symptoms in early development (rated retrospectively). We studied a group of children with mixed anxiety disorders. Although a ‘pure’ group of, for example, children with social anxiety disorders only may also be of interest, comorbidity among anxiety disorders is high and therefore ‘pure’ cases hardly exist in the real world of children seeking treatment for anxiety (e.g., Kendall et al. 2010 reported that almost 80 % of the anxiety disordered youth with a principal diagnosis of generalized anxiety disorder, separation anxiety disorder or social anxiety disorder, was also diagnosed with at least one of these three disorders).

## Method

### Participants

Forty-two children were referred to several mental health care centers in the Netherlands for treatment of clinical anxiety disorders. They participated in a study that compares treatment effectiveness for anxiety disorders in children with and without ASD. None of the children with anxiety disorders in the study had received an ASD diagnosis or were suspected of having ASD at intake. All of the clinically referred children were diagnosed with a DSM-IV anxiety disorder by the multi-disciplinary team of the mental health care centers. In addition, anxiety disorders were assessed with the Anxiety Disorder Interview Schedule—Child/Parent version (ADIS-C/P; Silverman and Albano 1996), which possesses good psychometric qualities (e.g., Silverman et al. 2001; Wood et al. 2002). The mean number of anxiety disorders in the clinical group was 3.52 (*SD* = 2.61). For an overview of the primary anxiety disorders and comorbid diagnoses of the clinically referred children, see Table 1 (a combined diagnosis of the ADIS-C/P was used).

**Table 1:** Primary anxiety disorder and comorbid disorders for the clinically anxious children

Primary disorder			Comorbid disorders (*n*)								
	*n*	*%*	SAD	SOC	SPH	GAD	OCD	PAN	AGOR	PTSD	MOOD
SAD	7	16.7	–	1	3	2	1	0	1	2	1
SOC	12	28.6	2	–	7	6	0	1	2	1	2
SPH	9	21.4	2	2	–	2	0	0	0	0	2
GAD	6	14.3	0	2	4	–	0	0	0	2	2
OCD	4	9.5	0	1	3	3	–	0	0	0	2
PAN	3	7.1	1	2	1	0	1	–	2	0	0
AGOR	1	2.4	0	1	1	1	0	0	–	1	0

*SAD* separation anxiety disorder, *SOC* social anxiety disorder, *SPH* specific phobia, *GAD* generalized anxiety disorder, *OCD* obsessive compulsive disorder, *PAN* panic disorder, *AGOR* agoraphobia, *PTSD* post traumatic stress disorder, *MOOD* mood disorder (dysthymic disorder/depressive disorder)

Typically developing children (*n* = 42) were recruited by graduate students via schools, daycare facilities, and convenience sampling. Children were excluded if they had a classified DSM-IV diagnosis, or if they were currently referred to mental health care centers for anxiety or other behavioral-emotional problems. Anxiety disorders (as measured with the ADIS-C/P), however, were present in five children (11.9 %): two had an ADIS-C/P diagnosis for generalized anxiety disorder, one for social anxiety disorder, one for separation anxiety disorder and one had an ADIS-C/P diagnosis for specific phobia. It was decided not to exclude those cases because: (a) anxiety disorders are very common in children and the rate found in this study is in accordance with the prevalence rates of anxiety disorders found in other studies (e.g., Costello et al. 2005), which strengthens our believe that the sample is representative for the population of typically developing children, and (b) the children were not clinically referred for their anxiety problems, nor did they seek any treatment for their anxiety problems following disclosure of the research diagnoses.

The group of children with anxiety disorders consisted of 21 boys and 21 girls with a mean age of 12.50 (*SD* = 2.90). The group of typically developing children consisted of 22 boys and 20 girls with a mean age of 11.38 (*SD* = 3.34). Groups did not differ with respect to gender, *X* ^*2*^[1] = 0.05; *p* = .827, or age, *F* [1, 82] = 2.71; *p* = .104.

### Instruments

#### Scared-71

The parent version of the Screen for Child Anxiety Related Emotional Disorders (SCARED-71; Bodden, Bögels and Muris 2009) was used in the current study to measure anxiety symptoms. The SCARED-71 is a questionnaire that consists of 71 items that have to be rated on a 3-point scale (almost never—sometimes—often). Besides calculating a total anxiety score, the 71 items can be grouped in various anxiety subscales measuring symptoms of panic disorder (13 items), social anxiety disorder (9 items), separation anxiety disorder (12 items), specific phobia (15 items), generalized anxiety disorder (9 items), obsessive compulsive disorder (9 items) and post traumatic stress disorder (4 items). Good reliability and validity is reported by Bodden and colleagues (2009).

#### ADI-R

The Autism Diagnostic Interview-Revised (ADI-R; Lord, Rutter, and Le Couteur 1994) is a semi-structured interview developed as a tool to diagnose ASD. The interview is conducted with the caregiver(s) who is asked about their child’s (a) current behavior and (b) behavior in the past (usually concerning the age period of 4–5 years). The behavioral items are grouped in three domains following the DSM-IV criteria for autistic disorder (APA 2000); reciprocal social interaction, communication, and restricted and repetitive behaviors. A diagnostic algorithm is applicable for the items that focus on behaviors in the past (i.e., all scores have to exceed certain thresholds) to establish a diagnosis of ASD. Psychometric properties of the ADI-R were investigated in the study of Lord et al. (1994) that found high intra-class correlations for the three domains, fair to good internal consistencies and adequate reliability over time. Further, specificity and sensitivity of the interview was found adequate (Lord et al. 1997). In the current study the items of the diagnostic algorithm were used (further referred to as ‘early ASD symptoms’). For several cases, the ADI-R was administered to the parents after their child received treatment for anxiety problems. Of note, no significant differences with respect to the scores for early ASD symptoms were found between those with an ADI-R report administered before or after treatment.

#### CSBQ

The Children’s Social Behavioral Questionnaire (CSBQ; Luteijn et al. 2002) was used in the present study to measure current ASD-like behaviors. The 49 items of the CSBQ were rated by the parents, who are asked to what extent each description applies to their child, on a three-point scale (does not apply—sometimes or somewhat applies—clearly or often applies). The items can be summed into a total score and into six subscales, namely (a) behaviors not tuned to situation, (b) withdrawal, (c) orientation problems, (d) difficulties understanding social information, (e) stereotyped behaviors, and (f) fear of and resistance to change. Psychometric properties (validity, internal consistency, inter-rater reliability and test–retest reliability) were studied in a large Dutch sample study, and the CSBQ was found a valid and reliable instrument. Furthermore, the CBSQ was found to discriminate groups of children with ASD (high-functioning autism, PDD-NOS) from clinical and non-clinical groups (Hartman, Luteijn, Serra and Minderaa 2006).

### Procedure

The study in which the clinically anxious children participated was approved by a medical ethics board. The comparison group of typically developing children was recruited in a later stage of the research, for which approval was given by an ethics committee of the University of Amsterdam. Written consent was obtained and assessments were carried out by the first author, several diagnosticians/psychologists and graduate students (under the supervision of the first author). Assessments took place at the families’ homes or at mental health care centers. All interview administers were extensively trained in the ADI-R by the first author, who is certified for administering the interview. The training consisted of role-plays, watching and coding several videotapes (including interviews with parents of who’s children were not diagnosed or suspected of ASD), and round-table discussions about coding and administration. All coders were trained to achieve at least 80 % agreement with the first author. Inter-rater reliability was assessed and was found fair to excellent (inter-correlation coefficients between the first author and the different coders ranged between .73 and .94).

### Data-Analysis

Because the vast majority of the ADI-R was administered to both parents (i.e., both mother and father were present during the interview), parental report about their child’s anxiety (SCARED-71) and ASD-like behaviors (CSBQ) were averaged (of note, reports between mothers and fathers correlated significantly for each subscale; *r* = .38–.88, all *p*’s < .05 for the different SCARED-71 subscales, and *r* = .49–.82, all *p*’s < .05 for the different CSBQ subscales). The SCARED-71 and CSBQ scores were aggregated in part because ADI-R interviews were administered to both parents in most families, leading to an integration of mother’s and father’s ratings of early ASD symptoms. Also, in general, aggregated scores have been found to increase the reliability of assessments (Bögels and van Melick 2004). If one parent report was missing, the other was used. In total two reports of the SCARED-71 (2.4 %) and five reports of the CBSQ were missing (5.9 %). Missing values were estimated with 2-way imputation (based on group mean and post-treatment assessment) and analyses were run twice (with and without missing values), however, results were similar. Variables were transformed into Z-scores to explore possible outliers. One case was identified as having an outlier for the CSBQ total score, one case for the ADI-R total score and the social domain, and one case was identified as having an outlier for the ADI-R repetitive domain (Transformed Z’s > 3.29; Tabachnick and Fidell 2001). To minimize the possible confounding effects of these outliers, we replaced the outliers by the sample mean with the addition of three standard deviations (Field 2009). In addition, we ran analyses with and without the transformed outliers, however, results were similar. Some (sub-)scales were not normally distributed. Therefore, we also applied non-parametric tests and analyses yielded similar results.

## Results

### Early ASD Symptoms (ADI-R)

Compared to typically developing children, children with anxiety disorders had a higher mean total score of the ADI-R as well as for all ADI-R domains (see Table 2). Of the clinically anxious children, 15 children (35.7 %) met at least one out of three ADI-R thresholds: three (7.1 %) met the threshold for the social domain, seven (16.7 %) for the communication domain, and nine (21.4 %) for the repetitive domain. One child (2.4 %) met thresholds for all three domains. Of the typically developing children, none met ADI-R thresholds for any domain. Exploratory post hoc analyses using Chi-squares revealed that children with anxiety disorders differed from typical developing children with respect to their scores for various items of the ADI-R (a p value of .01 was considered significant for these analyses in an effort to control for multiple comparisons; however, the exact p value for each item is given in Table 3). The most commonly endorsed ADI-R items (by 25 % or more) of the children with anxiety disorders were (in sequential order): spontaneous imitation, imaginative play with peers, imaginative play, compulsions/rituals, offering to share, social verbalization/chat, offering comfort, group play, conventional gestures, circumscribed interests, appropriateness of responses, interests in children, and response to approaches.

**Table 2:** ASD symptoms in children with anxiety disorders, and control children

	Anxiety		Control				
	*M*	*SD*	*M*	*SD*	*ES*	*F*	*p*
*Early ASD symptoms (ADI-R)*							
Total (range 0–74)	10.26	7.78	2.12	3.31	1.36	38.91	<.001**
Social (range 0–32)	4.48	4.46	1.43	2.61	0.83	14.64	<.001**
Communication (range 0–26)	4.31	3.61	0.60	1.19	1.38	40.06	<.001**
Repetitive behavior (range 0–16)	1.48	1.66	0.10	0.30	1.16	28.29	<.001**
*Current ASD-like behaviors (CSBQ)*							
Total score (range 0–98)	19.08	14.91	6.23	5.56	1.14	23.28	<.001**
(1) Behaviors not tuned to situation (range 0–22)	5.76	4.67	2.46	2.50	0.88	13.17	<.001**
(2) Withdrawal (range 0–24)	4.62	4.89	0.90	1.12	1.05	20.97	<.001**
(3) Orientation problems (range 0–16)	1.86	2.60	0.75	1.08	0.56	4.38	.013*
(4) Difficulty understanding social information (range 0–14)	2.95	3.13	1.60	1.73	0.54	5.08	.016*
(5) Stereotyped behaviors (range 0–16)	1.67	2.34	0.29	0.64	0.81	12.31	<.001**
(6) Fear of and resistance to changes (range 0–6)	2.23	1.98	0.25	0.58	1.36	39.80	<.001**

* *p* < .05; **^^ *p* < .01

*ES* Effect size (Cohen’s d)

**Table 3:** Exploratory post hoc analyses of the number of children with a 0, 1, or 2 score on ADI-R items representing early ASD symptoms: children with anxiety disorders compared to controls

	Anxiety			Controls			*X* ^2^	*p* ^a^
	0	1	2	0	1	2		
*Social domain*								
Use of other’s body	35	7	0	41	1	0	4.79	.057
Imaginative play with peers	24	6	12	40	2	0	16.80	<.001**
Direct gaze	33	7	2	39	3	0	3.50	.061
Social smiling	34	6	2	36	5	1	0.34	.558
Showing/directing attention	40	0	2	42	0	0	2.05	.494
Offering to share	29	6	7	35	3	4	2.36	.124
Seeking to share enjoyment	37	2	3	41	1	0	2.87	.202
Offering comfort	30	10	2	38	4	0	4.94	.026
Quality of social overtures	39	2	1	42	0	0	3.11	.241
Range of facial expressions	32	8	2	42	0	0	11.35	.001*
Inappropriate facial expressions	36	6	0	41	1	0	3.90	.109
Appropriateness of responses	31	7	4	40	1	1	7.37	.007*
Interest in children	31	9	2	34	5	3	0.61	.434
Response to approaches	31	9	2	38	3	1	3.98	.046
Group play	30	11	1	35	6	1	1.70	.192
Friendships	38	1	3	40	1	1	0.72	.676
*Communication domain*								
Stereotyped utterances	34	8	0	42	0	0	8.84	.005*
Social verbalization/chat	29	5	8	40	2	0	9.82	.002*
Reciprocal conversation	32	7	3	42	0	0	11.35	.001*
Inappropriate questions	39	0	3	38	4	0	0.16	1.00
Pronominal reversal	36	6	0	42	0	0	6.46	.026
Neologisms	34	7	1	42	0	0	8.84	.005*
Pointing to express interest	34	4	4	38	1	3	1.56	.212
Nodding	34	6	2	41	1	0	6.10	.029
Head shaking	34	6	2	42	0	0	8.84	.005*
Conventional gestures	30	7	5	39	1	2	6.57	.010
Spontaneous imitation	22	9	11	41	1	0	22.92	<.001**
Imaginative play	25	10	7	38	4	0	10.73	.001*
Imaginative social play	33	4	5	41	1	0	7.27	.007*
*Repetitive domain*								
Verbal rituals	40	2	0	42	0	0	2.05	.494
Unusual preoccupations	41	1	0	42	0	0	1.01	1.00
Circumscribed interests	30	11	1	41	1	0	11.01	.001*
Repetitive use of objects	40	1	1	42	0	0	2.05	.494
Compulsions/rituals	26	8	8	41	1	0	16.59	<.001**
Unusual sensory interests	38	4	0	42	0	0	4.20	.116
Hand/finger mannerisms	32	9	1	40	2	0	6.22	.013
Other complex mannerisms	38	4	0	42	0	0	4.20	.116

* *p* < .01; ** *p* < .001

^a^For analyses, ADI-R item scores of 1 and 2 were collapsed and the corresponding *p* value of Fisher’s Exact Test was reported if the expected cell count of at least one cell was less than 5

**Fig. 1:**
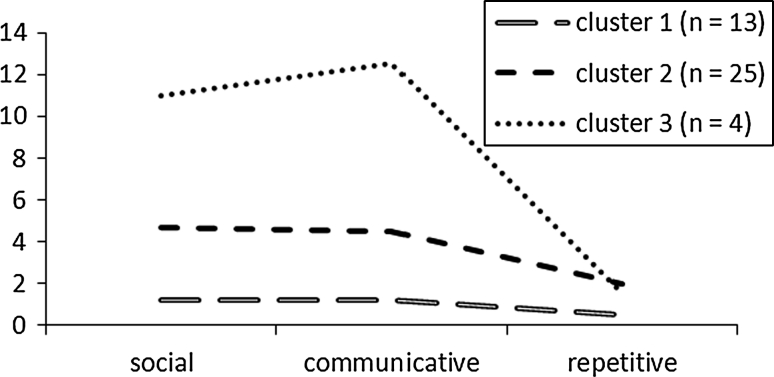
Profiles of the three clusters of children with anxiety disorders for their mean ADI-R algorithm scores in the early childhood social, communication, and repetitive domains

A cluster analysis was run on the scores of the three ADI-R domains (social, communication and repetitive behavior) using the sample of children with anxiety disorders only (*n* = 42) to examine how many children would display an ASD-like profile. Ward’s hierarchical cluster analysis using squared Euclidean distances as the distance measure was used. To establish the number of clusters, the percentage change in agglomeration coefficients and inspection of the dendrogram was used. Using this approach, three clusters were identified; all clusters differed significantly with respect to the mean ADI-R social and communication scores, and cluster one and cluster two differed with respect to the mean ADI-R repetitive scores (see Fig. 1). The first cluster (*n* = 13) is characterized by low scores in every ADI-R domain. The second cluster (*n* = 25) is characterized by medium scores in the social and communicative domain and a somewhat higher score in the repetitive domain. Finally, the third cluster (*n* = 4) is characterized by a PDD-NOS-like profile: high scores on both the social and communication domain, and medium scores for the repetitive domain.

None of the children in the first cluster met any ADI-R threshold. Of the second cluster, none of the children met the ADI-R threshold for the social domain, however three children (12.0 %) met the threshold for the communication domain and eight children (32.0 %) met the threshold for the repetitive domain. Most commonly endorsed problems (ADI-R items) for the children in this cluster were: spontaneous imitation (score 0 = 40 %; score 1 = 28 %; score 2 = 32 %), imaginative play with peers (score 0 = 52 %; score 1 = 16 %; score 2 = 32 %), compulsions/rituals (score 0 = 52 %; score 1 = 24 %; score 2 = 24 %), offering to share (score 0 = 56 %; score 1 = 16 %; score 2 = 28 %), and imaginative play (score 0 = 60 %; score 1 = 20 %; score 2 = 20 %). With respect to the children in the third cluster it was found that three children (75.0 %) met the threshold for the social domain, all four children (100.0 %) met the cutoff for the communication domain and one child (25.0 %) met the cutoff for the repetitive domain. Most commonly endorsed ADI-R items for this cluster concerned problems with: imaginative play with peers (score 1 = 25 %; score 2 = 75 %), social verbalization/chat (score 1 = 25 %; score 2 = 75 %), conventional gestures (score 1 = 25 %; score 2 = 75 %), reciprocal conversation (score 1 = 50 %; score 2 = 50 %), and group play (score 1 = 75 %; score 2 = 25 %).

### Current ASD-Like Behaviors (CSBQ)

Compared to typically developing children, those with anxiety disorders were found to have significantly higher mean scores for ASD-like behaviors in general (CSBQ total score) and for all subscale scores (see Table 2). Thirteen children with anxiety disorders (31.0 %) had scores that fell in the ASD range. None (0.0 %) of the typical developing children had scores falling in the ASD range.

### Correlations

For the clinically referred children only (*n* = 42), correlations were calculated between early ASD symptoms (ADI-R), current ASD-like behaviors (CSBQ), and anxiety symptoms (SCARED-71). The correlation between early ASD symptoms and current ASD-like behaviors was .36 (*p* = .020), indicating that higher scores of early ASD symptoms were related to higher scores of current ASD-like behaviors. For current ASD-like behavior (CSBQ), correlations with the following SCARED-71 subscales were significant (in sequential order from lowest to highest); specific phobia, separation anxiety disorder, generalized anxiety disorder, panic disorder, and social anxiety disorder (varying from .38 to .59; see Table 4). All correlations were positive, indicating that higher current ASD-like behaviors were related to higher anxiety symptoms. For early ASD symptoms only one correlation, with symptoms of social anxiety disorders, was found significant (Table 4). Although this correlation did not statistically differ from the second highest correlation, the magnitude of this correlation was medium (*r* = .38), while the other correlations were found to be small (*r* ≤ .24).

**Table 4:** Correlations between current anxiety symptoms (SCARED-71) and early ASD symptoms (ADI-R), and current ASD-like behavior (CSBQ) in clinically referred children with anxiety disorders (n = 42)

	SCARED-71						
	PAN	GAD	SOC	SAD	OCD	PTSD	SPH
Early ASD symptoms (ADI-R)	.06	.02	.38*	.24	.14	.10	.24
Current ASD-like behavior (CSBQ)	.58**	.50**	.59**	.49**	.29	.21	.38*

*PAN* panic disorder, *GAD* generalized anxiety disorder, *SOC* social anxiety disorder, *SAD* separation anxiety disorder, *OCD* obsessive compulsive disorder, *PTSD* post traumatic stress disorder, *SPH* specific phobia

* *p* < .05; ** *p* < .01

## Discussion

This study explored autistic symptoms and ASD-like behaviors in children with anxiety disorders. The main results were: (a) Parents reported that their school-aged children with anxiety disorders had significantly more ASD symptoms in early childhood than typically developing children; (b) over one-third of children with an anxiety disorder, but no known history of ASD, exceeded at least one of the three ADI-R thresholds for early childhood clinically significant ASD symptoms; and (c) early ASD symptoms were found to be related to current ASD-like behaviors as well as current symptoms of social anxiety disorder.

It was found that ASD symptoms seem to be present early in life (based on retrospective reports of parents) in some children who have developed clinical anxiety but who are not recognized by professionals as having current ASD. Cluster analysis revealed that a small number of children (*n* = 4) showed a classic, if moderate, ASD-like profile (high scores on both the social and communication domains, as well as moderate repetitive behaviors), while the majority of the children with anxiety disorders (*n* = 25) were characterized by a milder ASD-like early childhood phenotype. Commonly endorsed items for the latter group included items such as early childhood impairments in spontaneous imitation, imaginative play (with peers), and offering to share, but at a low-severity level. Interestingly, the most commonly endorsed symptoms, and significant differences between clinically anxious and controls, were found for items in the communication domain. Similarly, the study of Hallett et al. (2010) found that early communication difficulties in children from the general population contributed more strongly to internalizing traits in later life than social difficulties and repetitive behaviors.

With the exception of symptoms of obsessive compulsive disorder and post traumatic stress disorder, all other anxiety symptoms were found to be associated with current ASD-like behaviors. In contrast, only one (symptoms of social anxiety disorder) was found to be associated with early ASD symptoms (as measured with the ADI-R). It should be noted that the instruments to assess anxiety symptoms (SCARED-71) and current ASD-like behaviors (CSBQ) are more similar (likely resulting in higher correlations between these two instruments) compared to the instrument used to assess early ASD symptoms (ADI-R). However, it may also be that children with a moderate or high degree of ASD symptoms early in life, may be more prone to develop (symptoms of) some (social anxiety) rather than other anxiety disorders. In addition, although different instruments were used to assess early ASD symptoms and current ASD-like behaviors, evidence for moderate ASD-symptom stability was found in the current study (i.e., the correlation between early ASD symptoms and current ASD-like behaviors was found to be medium and significant, *r* = .36), however, it was not as large as in other studies that used the same instrument to asses ASD traits over time (e.g., the correlation between ASD traits over a 5-year period of time was found to be .59 for boys and .55 for girls in the study of Hallett et al. 2010).

With respect to current ASD-like behaviors, this study found almost one-third (31.0 %) of the clinically anxious sample falling in the ASD range on the current ASD-like behavior instrument (CSBQ). This finding is in accordance with previous studies (Towbin et al. 2005; Pine et al. 2008), although Pine and colleagues reported a somewhat lower percentage for the children with anxiety disorders (i.e., 4.0–24.6 %). The relatively high percentage may partly be explained by symptom overlap in the instrument used to assess current ASD-like behaviors (e.g., items of the CSBQ such as ‘makes little eye-contact’ or ‘does not initiate to play with others’ may very well be present in socially anxious children, while those behaviors may not necessarily be attributable to ASD). Nevertheless, it may be important in clinical practice to explore current ASD-like behaviors in children with anxiety disorders as the study of Puleo and Kendall (2011) found that the presence of ASD traits in anxious children not identified to have ASD may have consequences for treatment choice. That is, children with moderate ASD traits were found to profit more from family oriented cognitive behavioral therapy (CBT) compared to individual CBT (Puleo and Kendall 2011). Note also that Sofronoff et al. (2005) found that for the treatment of anxiety in children with Asperger, active parental involvement enhanced the effects of this intervention. Contradictory, in typically developing children with anxiety disorders an additional effect of a family component is not always found (e.g., In-Albon and Schneider 2007; Bodden et al. 2008); however, those studies did not explore the possible role of ASD traits.

Finally, limitations of the study have to be addressed. The first limitation is that ASD symptoms in early development were assessed retrospectively, and although the ADI-R has good psychometric properties (e.g., Lord et al. 1994), recall bias cannot be excluded. In addition, it cannot be ruled out that recall-bias is different in the two groups. That is, it might be that parents of a child with an anxiety disorder report more negative aspects of their child’s development (e.g., due to the stress associated with having a child with an anxiety disorder) compared to parents of control children. However, it was also found that the early ASD scores from parents who were interviewed after their child received treatment (perhaps being more in a positive state of mind because of successful treatment outcome) did not differ from the scores of parents interviewed before their child received treatment. Furthermore, the distinct clusters of early ASD symptoms revealed in the cluster analysis suggest that only a minority of children were reported to have a pattern of significant ASD symptomatology in early childhood, suggesting at the least that such a recall bias could not have affected the parents of children with anxiety disorders uniformly. Nonetheless, as recall bias cannot be ruled out when using a retrospective measure, a potential supplemental research strategy for future studies would be to ask the parents to provide home videotapes about their child’s early development, and then rate the ASD-related behaviors (note that ASD-research already made use of such an approach; e.g., Osterling et al. 2002; Werner and Dawson 2005).

A second limitation of the study is that we could not establish temporal precedence for we did not assess anxiety early in life. It may well be that anxiety disorders were already present early in life and may have not been noticed or treated until later in life. For example, social anxiety disorder may have its onset early in development and behavioral inhibition is thought to be a precursor (e.g., Bögels et al. 2010; Rapee and Spence 2004). Behavioral inhibition is viewed as a temperament style that consists of a pattern of behaviors such as avoidance, withdrawal, shyness, and reticence that manifest in response to novelty or unfamiliarity (Hirshfeld-Becker et al. 2007). Although there may be some overlap between items of the ADI-R and behavioral inhibition under select novel conditions (e.g., items of the ADI-R asking about ‘seeking to share one’s enjoyment with others’ or ‘initiating social talk’), the ADI-R primarily includes items that share (very) little overlap with behavioral inhibition (e.g., ‘imaginative play’ or ‘facial expressions’). In addition, post hoc analyses revealed significant differences between the clinically anxious children and the typically developing children for such non-overlapping items, suggesting that the early life ASD symptoms found in clinically anxious children are not solely attributed to the diagnostic overlap with other constructs like behavioral inhibition. However, considering the cross-sectional nature of this study, results about the relation between elevated ASD symptoms in early development and anxiety disorders later in life should be interpreted with caution and viewed with the perspective that these are preliminary findings that should be further explored.

Third, relatively few children (*n* < 10) were diagnosed with obsessive compulsive disorder, panic disorder, agoraphobia and post traumatic stress disorder. It is possible that this confounded the results. For example, certain autistic-like behaviors are common in children with obsessive compulsive disorder (e.g., Ivarsson and Melin 2008), and autistic-like behaviors in adults were found to be more common in those with obsessive compulsive disorder than in those with social anxiety disorder (Bejerot and Mörtberg 2009). It could be that differences between the clinical group and controls would have been even larger if more cases of obsessive compulsive disorder were included. Larger sample sizes of ‘pure’ cases (e.g., children with just social anxiety disorder or obsessive compulsive disorder) would in some ways have been preferable to explore the specific associations between anxiety disorders and ASD symptoms early in life. However, as noted in the introduction, clinically anxious children are often diagnosed with multiple anxiety disorders (Kendall et al. 2010), making such comparisons complicated and unrepresentative of treatment-seeking children with clinical anxiety.

A final limitation is that we did not have IQ data available for the two groups and that the clinically anxious children consisted of a very heterogeneous sample. That is, our sample of children with anxiety disorders consisted of a variety of anxiety disorders and most of the children had multiple anxiety disorders. On the other hand, all were highly verbal and able to respond appropriately to our anxiety disorders diagnostic interview, denoting roughly age-appropriate levels of verbal communication.

Despite the addressed limitations, the relationship between anxiety and ASD is interesting and warrants further investigation. Not only are anxiety disorders highly prevalent and perhaps somewhat (phenomenologically) endemic in individuals with ASD, but also—as found in this and other studies—early ASD symptoms and current ASD-like behaviors are significantly more prominent in children with anxiety disorders. Interesting, the two disorders may share some deficits in the same brain region. For example, for both children with ASD as well as clinically anxious children, anxiety symptoms were found to be associated with abnormalities in the functioning of the amygdala (Thomas et al. 2001; Juranek et al. 2006). In addition, similar genetic markers are found for anxiety in children with autism and typically developing children (e.g., Gadow et al. 2008). However, results concerning the amygdala are inconclusive, other brain areas (as well as other biomarkers) are found to be abnormal in ASD (e.g., Brambilla et al. 2003; Hughes 2009), and apparent similarity may disguise underlying differences. Gregory and Eley (2007) concluded that environmental factors are at least equally important as genetic factors for the differentiation of individual anxiety levels in children. Furthermore, Hallett et al. (2009) found little evidence for genetic influences with respect to the phenotypic correlation between autistic traits and internalizing traits in the general population, and propose that anxiety may be a response to autistic-like difficulties. Likely, the stress experienced by many children with moderate to high ASD symptoms promotes anxiety and mood disorders (Wood and Gadow 2010), however, more empirical research is needed. Although part of the results (found here and elsewhere) may be conflicted by diagnostic overlap between ASD and anxiety, the present findings do warrant further investigation of the relationship between the two disorders, and more specifically of the role of ASD symptoms in the development, maintenance, and treatment of anxiety disorders.
